# Histomorphometric case-control study of subarticular osteophytes in patients with osteoarthritis of the hip

**DOI:** 10.1186/s12891-020-03648-w

**Published:** 2020-10-06

**Authors:** Rasmus Klose-Jensen, Andreas Wiggers Nielsen, Louise Brøndt Hartlev, Jesper Skovhus Thomsen, Lene Warner Thorup Boel, Mogens Laursen, Kresten Krarup Keller, Ellen-Margrethe Hauge

**Affiliations:** 1 Department of Rheumatology, Aarhus University Hospital, Palle Juul-Jensens Boulevard 45, 8200 Aarhus, Denmark; 2 Department of Clinical Medicine, Aarhus University, Aarhus, Denmark; 3 Department of Clinical Medicine, Randers Regional Hospital, Randers, Denmark; 4 Department of Biomedicine – Anatomy, Aarhus University, Aarhus, Denmark; 5 Institute of Forensic Medicine, Aarhus University, Aarhus, Denmark; 6 Orthopaedic Surgery Research Unit, Aalborg University Hospital, Aalborg, Denmark; 7 Diagnostic Centre, Silkeborg Regional Hospital, Silkeborg, Denmark

**Keywords:** Osteoarthritis, Femoral head, Osteophytes

## Abstract

**Objective:**

The objective of this cross-sectional case-control study was to determine the prevalence and size of marginal and subarticular osteophytes in patients with osteoarthritis (OA), and to compare these to that of a control group.

**Design:**

We investigated femoral heads from 25 patients with OA following hip replacement surgery, and 25 femoral heads from a control group obtained *post-mortem*. The area and boundary length of the femoral head, marginal osteophytes, and subarticular osteophytes were determined with histomorphometry. Marginal osteophytes were defined histologically as bony projections at the peripheral margin of the femoral head, while subarticular osteophytes were defined as areas of bone that expanded from the normal curvature of the femoral head into the articular cartilage.

**Results:**

The prevalence of OA patients with marginal- and subarticular osteophytes were 100 and 84%, respectively. Whereas the prevalence of the participants in the control group with marginal- and subarticular osteophytes were 56 and 28%, respectively.

The area and boundary length of marginal osteophytes was (median (Interquartile range)) 165.3mm^2^ (121.4–254.0) mm^2^ and 75.1 mm (50.8–99.3) mm for patients with OA compared to 0 mm^2^ (0–0.5) mm^2^ and 0 mm (0–0.5) mm for the control group (*P* <  0.001). For the subarticular osteophytes, the area and boundary length was 1.0 mm^2^ (0–4.4) mm^2^ and 1.4 mm (0–6.5) mm for patients with OA compared to 0 mm^2^ (0–0.5) mm^2^ and 0 mm (0–0.5) mm for the control group (*P* <  0.001).

**Conclusion:**

As expected, both marginal- and subarticular osteophytes at the femoral head, were more frequent and larger in patients with OA than in the control group. However, in the control group, subarticular osteophytes were more prevalent than expected from the minor osteophytic changes at the femoral head margin, which may suggest that subarticular osteophytes are an early degenerative phenomenon that ultimately might develop into clinical osteoarthritis.

## Background

Osteoarthritis (OA) is the most common joint disease, and OA of the hip afflicts 3–6% of the population over 50 years [1]. The disease is characterised by loss of articular cartilage, sclerosis of the subchondral bone and formation of osteophytes [2]. Importantly, it has been shown that subchondral bone sclerosis and formation of marginal osteophytes at the knee could be detected before the thickness of the articular cartilage changes and the joint space narrows [3, 4]. Marginal osteophyte formation is thought to occur by the proliferation of periosteal- and synovium-derived mesenchymal stem cells differentiating into chondrocytes that form cartilage and through endochondral ossification leads to bony projections [5]. However, the formation of marginal osteophytes is likely not the only example of bone formation in OA. It has been hypothesised, that microcracks in the subchondral bone plate and the calcified cartilage may reactivate the secondary ossification centre and lead to loss of cartilage through endochondral ossification [6]. Using quantitative backscattered electron imaging, Ferguson et al. found high density mineralised protrusions (HDMP) at the mineralising front of calcified cartilage in femoral heads from patients with OA [7]. These HDMP have also been found in the knee of patients with OA, using magnetic resonance imaging (MRI) and micro-computed tomography [8]. The HDMP emerged from the calcified cartilage and subchondral bone junction and could extend up to two-thirds of the articular cartilage thickness, resulting in cartilage degeneration [9]. The mechanism for this cartilage degeneration is currently unknown, but HDMP could perhaps be associated with endochondral ossification and subarticular osteophyte formation. McCauley et al. showed that subarticular osteophytes in the knee visualised with MRI were associated with articular cartilage defects [10]. Similarly to HDMP, these subarticular osteophytes were defined using MRI [11]. At present, it is unknown whether HDMP transforms into subarticular osteophytes or whether subarticular osteophytes actually are HDMP. Until now, subarticular osteophytes have not been investigated with quantitative histomorphometric methods.

We acknowledge that subarticular osteophytes exist and might be part of the early pathological changes in patients with osteoarthritis. To further elucidate this hypothesis, we quantified the boundary length and the area of the entire femoral head, the marginal osteophytes and the subarticular osteophytes in both patients with OA and a control group with similar sex and age distribution.

## Materials & methods

The article was designed in accordance with Strengthening the Reporting of Observational Studies in Epidemiology (STROBE) checklist [12].

### Study design and ethical considerations

The study was a cross-sectional histomorphometry study of marginal- and subarticular osteophytes in the femoral heads obtained from 25 patients with OA and 25 deceased subjects with similar sex and age distribution.

The arthritic femoral heads were obtained from patients with primary hip OA, who underwent total hip replacement surgery at the Department of Orthopaedics, Farsoe Hospital, Denmark. Prior to surgery, clinical assessment of OA based on pain, stiffness, and physical function, Western Ontario and McMaster Universities Arthritis Index (WOMAC, VAS3.1) [13] scheme was fulfilled. The WOMAC questionnaire is based on 24 component item scores, and the scores were normalised to 100 mm using the following correction factors: the sum of pain score × 0.5, the sum of stiffness score × 0.2, and the sum of physical function score × 0.059 [13]. The radiographic classification of OA in the hip joint was evaluated using the Kellgren-Lawrence grading scale, which grades OA between 0 to 4 depending on severity [14].

Macroscopically normal femoral heads from the control group were obtained at autopsy from individuals who had died suddenly from accidents or acute diseases at the Department of Forensic Medicine, Aarhus University, Denmark.

The Ethics Committee of Medical Research in Denmark Region (J. no. 10776) and The Danish Data Protection Agency (J.nr: 2003-41-3447) approved the study. Written and informed consents were obtained from the patients with OA before their hip replacement surgery.

### Participant inclusion and exclusion criteria

#### Patients with osteoarthritis

Inclusion criteria for the OA patients were the fulfilment of the American College of Rheumatology clinical and radiographic criteria for OA [15] and a referral to total hip replacement surgery. Exclusion criteria were known bone metabolic diseases, diabetes mellitus, malignant diseases, secondary OA or other joint diseases.

#### Control group

Inclusion criteria were newly deceased subjects with macroscopically healthy femoral heads. The participants from the control group were excluded if they had a history of high-energy pelvic trauma, known diagnosis of bone metabolic disease, diabetes mellitus, malignant diseases or other joint diseases were also excluded.

#### Sample size

This is the first study to quantify subarticular osteophytes by histomorphometry. Therefore, no data is available on relevant populations for which sample size calculations can be made. We used the same study population, which we have used previously for investigating bone turnover in relation to overlying cartilage deterioration [16].

### Outcome measures

#### Processing of tissue

Immediately after the removal, the femoral heads were fixed in 70% ethanol and then processed according to stereological sampling of vertical uniform random sections [17], previously described in detail [16, 18–20]. In brief: the entire femoral heads were rotated around a vertical axis, which was perpendicular to the anatomical top of the femoral head. After choosing a random starting point, the femoral head was sawed using a diamond precision-parallel saw (Exakt Apparatebau, Norderstedt, Germany) into 7-mm-thick parallel slices, which were halved, and alternating left and right half slices were randomly selected for the following microscopic evaluation [17, 18]. Depending on the size of the femoral head, a total of five to seven 7-mm-thick halved parallel slices were collected from both patients with OA and controls. Each of the five to seven 7-mm-thick halved parallel slices were embedded undecalcified in methylmethacrylate and cut into 7-μm-thick histological sections using a Jung model K microtome (R Jung, Heidelberg, Germany) equipped with a tungsten microtome knife. The sections were mounted and stained with Masson-Goldner trichrome [18].

#### Definition of tissue structures

**Fig. 1:**
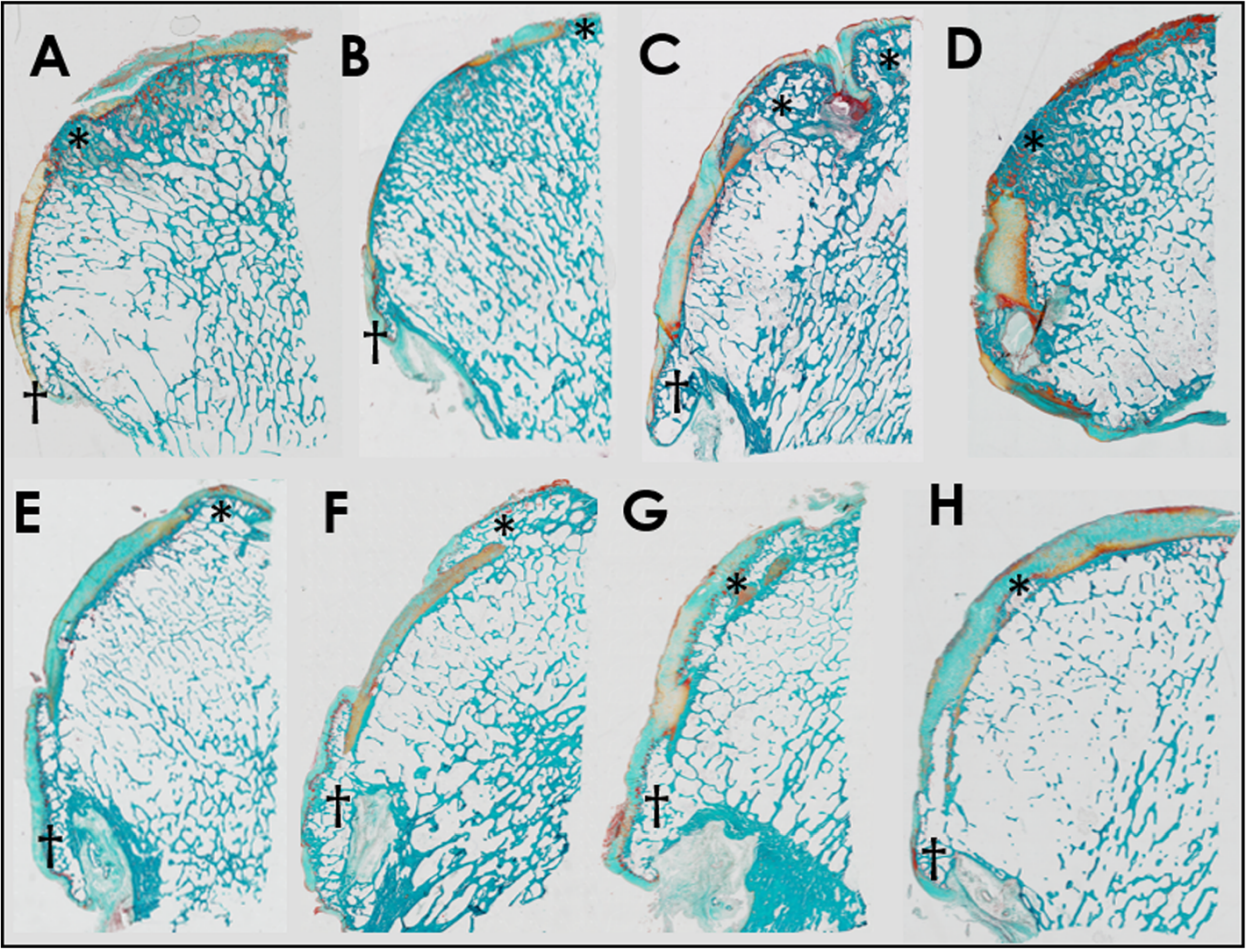
Masson-Goldner trichrome stained 7-μm-thick tissue section of a halved femoral heads. **a** 59-year-old male osteoarthritis (OA) patient. **b** 62-year-old female OA patient. **c** 56-year-old male OA patient. **d** 73-year-old female OA patient. **e** 67-year-old male OA patient. **f** and **g** 61-year-old male OA patient. **h** 66-year-old female control. An asterisk denotes the subarticular osteophytes. A cross denotes the marginal osteophytes

Marginal osteophytes were defined as bony projections at the peripheral margin of the femoral head (5) (Fig. 1). Subarticular osteophytes were defined as areas of bone, which expanded from the normal curvature of the femoral head into the cartilage, and which was not located in relation to the peripheral margin of the femoral head or the fovea capitis [21] (Fig. 1). The fovea capitis was excluded in the present study.

#### Histomorphometry

Data were collected using a light microscope (Nikon Eclipse 80i, Tokyo, Japan) equipped with a motorised specimen stage (Prior Proscan 11 TM, Rockland, MA, USA), a microcator (Heidenhain MT 1201, Traunreut, Germany), and a digital video camera (OlympusDP72, Tokyo, Japan) connected to a PC running the newCAST interactive stereology software (v. 3.4.1.0, Visiopharm, Hørsholm, Denmark). Sampling regions were automatically aligned in newCAST. Test points and test lines were superimposed on the digital images of the tissue sections and viewed on the PC monitor at a total magnification of × 121.64. For each of the 7-mm-thick parallel tissue sections, the profile number of subarticular and marginal osteophytes were counted, and the area and boundary length of the femoral head, subarticular- and marginal osteophytes were estimated.

The total area (*Ar*) of the femoral head, subarticular- and marginal osteophytes were estimated with point counting using all 7-mm-thick halved parallel tissue section from each individual femoral head. For estimations of the area of the subarticular and marginal osteophytes, a grid with an area per point of 0.49 mm^2^ was used. For estimations of the area of the femoral head_,_ a grid with an area per point of 2.93 mm^2^ was used.

$$ Ar=a(p)\times \sum \limits_{i=1}^np $$

Where *n* is the number of sections, ${\sum}_{i=1}^np$ is the total number of test points hitting the structure of interest and *a(p)* is the area per test point [22].

The total boundary length (Bd) of the femoral head, subarticular- and marginal osteophytes from the five to seven 7-mm-thick halved parallel tissue section, were estimated using a line probe with an area per length of 0.297 mm.

$$ Bd=\pi /2\times \sum \limits_{i=1}^nl\times a(l) $$

Where π/2 is a constant used for sine-weighted test-lines, *n* is the number of sections, ${\sum}_{i=1}^nl$ is the total number of intersections between the tissue surface and the sine-weighted test-line grid, and *a(l)* is the area per test line length of the line-grid superimposed on the tissue section [22].

### Statistical methods

Data were analysed using STATA 12 (StataCorp LP, College Station, TX, USA). Normal distribution of the data was investigated with Q-Q plots and histograms. Normally distributed data are presented as mean (95% confidence interval), and differences between the two groups were tested for statistical significance using Student’s *t*-test. Data, which was not normally distributed, are presented as median (Interquartile range (IQR)), differences between the two groups were tested for statistical significance using the Mann-Whitney U test. Coefficient of variation (CV) was calculated as the standard deviation of the two repeated measurements divided by the subject mean in order to investigate the intraobserver reliability of the measures. The results were considered statistically significant at *P* <  0.05.

## Results

Patients demographics are shown in Table 1.

**Table 1:** Participants demographics

	Control (*n* = 25)	Osteoarthritis (*n* = 25)	*p*-value
Female (%)	52	52	1.000
Age (years, mean ± SD)	61.5 ± 7.8	64.4 ± 7.2	0.175
Male	62.0 ± 8.5	64.3 ± 6.7	0.466
Female	61.0 ± 7.4	64.5 ± 7.8	0.258
Kellgren-Lawrence grade (mean ± SD)	–	3.8 ± 0.4	–
WOMAC Normalized NRS 3.0 (mm, mean ± SD)			
Pain	–	50 ± 21	–
Stiffness	–	56 ± 29	–
Physical Function	–	44 ± 14	–

*BMI* Body Mass Index, *WOMAC* The Western Ontario and McMaster Universities Arthritis Index

**Fig. 2:**
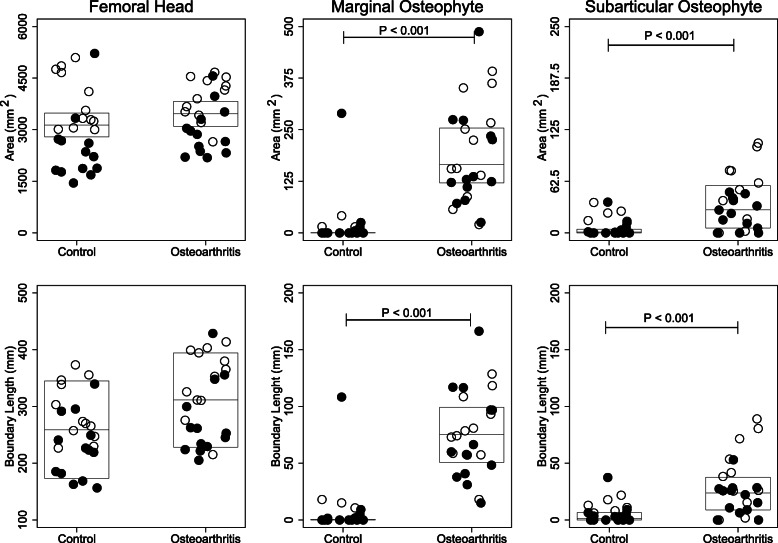
*Femoral Head:* Each open/black dot represents the total area or boundary length of the femoral heads for a male/female subject. The horizontal line indicates the mean of the control group and the patients with osteoarthritis, while the boxes represent the 95% confidence interval. Student’s *t*-test was used to test for statistical significance. *Marginal- and Subarticular osteophytes:* Each open/black dot represents the total area or boundary length of marginal- or subarticular osteophytes for a male/female subject. The horizontal line indicates the median of the control group and the patients with osteoarthritis, while the boxes represent the interquartile range. Mann–Whitney U test was used to test for statistical significance. *P*-value < 0.05 was considered significant

The median profile number (Table 2), area and boundary length for both marginal- and subarticular osteophytes were significantly larger in the patients with OA than in the control group (Fig. 2). The prevalence of subjects with marginal- and subarticular osteophytes were significantly larger in the patients with OA than in the control group (Table 2). In addition, 19 of the 25 (79%) patients with OA had more than one profile number of subarticular osteophytes, whereas in the control group only 7 of the 25 (28%) participants had more than one profile number of subarticular osteophytes (*p* = 0.001). The high median profile number and boundary length of subarticular osteophytes indicate an enlarged surface area of the femoral heads.

**Table 2:** Marginal- and subarticular osteophytes

	Control (*n* = 25)	Osteoarthritis (*n* = 25)	*P*-Value
*Marginal osteophytes*			
Prevalence of participants with marginal osteophytes, n (%)	7 (28)	25 (100)	< 0.001
Profile number of marginal osteophytes, number	16	133	< 0.001
Marginal osteophytes, median profile number (IQR)	0 (0 to 1)	5 (5 to 6)	< 0.001
*Subarticular osteophytes*			
Prevalence of participants with subarticular osteophytes, n (%)	14 (56%)	21 (84%)	< 0.001
Profile number of subarticular osteophytes, number	26	82	0.014
Subarticular osteophytes, median profile number (IQR)	3 (2 to 4)	5 (5 to 6)	< 0.001

*IQR* Interquartile range

In the patients with OA, 83% of the subarticular osteophytes was superficially covered with articular cartilage. The remaining 17% had a denuded bone surface. In the control group, 96% of the subarticular osteophytes were superficially covered with articular cartilage. The remaining 4% had a denuded bone surface. The proportion of subarticular osteophytes with denuded bone did not differ between patients with OA and the control group (*p* = 0.082).

The prevalence of OA patients with marginal osteophytes was significantly higher than the prevalence of OA patients with subarticular osteophytes (100% versus 84%, *p* = 0.037). Conversely in the control group, the prevalence of subjects with subarticular osteophytes was significantly higher than the prevalence of subjects with marginal osteophytes (56% versus 28%, *p* = 0.045).

For the femoral head, marginal osteophytes and for the subarticular osteophytes the CV were 1.3, 9.4 and 28.4%, respectively.

## Discussion

In this cross-sectional study, we found that subarticular osteophytes protruding from the normal curvature of the femoral head into the articular cartilage were a common feature of patients with OA. Surprisingly, the control group with macroscopically healthy femoral heads also had subarticular osteophytes, but they were much less common and smaller than in patients with OA. Marginal osteophytes are a hallmark of osteoarthritis. This was clearly apparent in the present study as the patients with OA had larger and more frequent marginal osteophytes than the control group.

To our knowledge, subarticular osteophytes have not previously been quantified using histology. McCauley et al. investigated central osteophytes from 200 patients who were referred for MRI of the knee [10]. In that study, central osteophytes were defined as focal excrescences that extended from the cortical surface and were surrounded by articular cartilage on all sides. This definition is similar to the definition of subarticular osteophytes used in the present study. McCauley et al. found that subarticular osteophytes were associated with more articular cartilage defects and meniscal tears. Increased weight and age are known to predispose to osteoarthritis [23, 24], and the patients with subarticular osteophytes were also significantly older and had a higher Body Mass Index than patients without subarticular osteophytes [10]. The present study only investigated the femoral head. However, other studies have shown that central osteophytes also occur in the acetabulum, and the formation of central osteophytes occurred prior to joint space narrowing [25] according to Tönnis radiographic classification of hip OA [26].

In the present study, the subarticular osteophytes were slightly more common than marginal osteophytes in the control group. It has been shown that bone growth, in the form of marginal osteophytes, occurs before a loss of articular cartilage could be detected on conventional radiographs [3]. Therefore, it is not unreasonable to suggest that subarticular osteophytes also could be an early degenerative phenomenon which may occur before any loss of articular cartilage is evident, particularly as the size and frequency of subarticular osteophytes were greater in patients with OA than in the control group. Although the difference was not statistically significant, 17% of the subarticular osteophytes had denuded bone in patients with OA compared to 4% of the control group indicating that the subarticular osteophytes form at the calcified cartilage invading the articular cartilage. Currently, the formation of marginal osteophytes, joint space narrowing, and subchondral bone sclerosis are the only features determined in radiographical OA assessment. However, these features may not be sufficiently sensitive to detect OA at an early stage, or sufficiently sensitive to change over time. Quantitative assessment of early osteoarthritic changes is necessary for investigating future medical therapies of OA. Therefore, we suggest that the subarticular osteophytes investigated in the present study might be an OA biomarker using quantitative computed tomography.

**Fig. 3:**
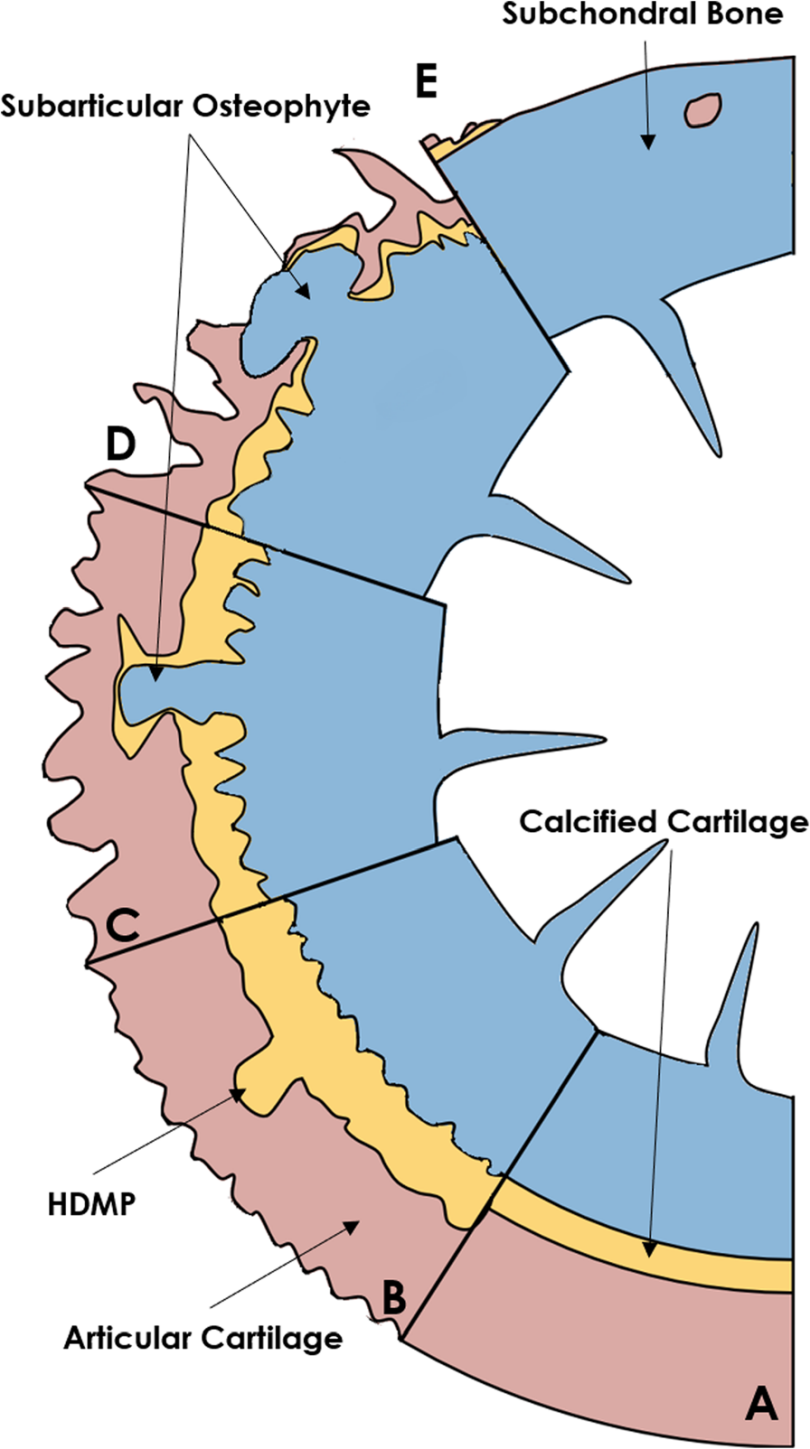
Schematic illustration of the hypothesised growth of subarticular osteophytes into articular cartilage. **a** Healthy joint surface with articular cartilage, calcified cartilage and subchondral bone. **b** High density mineralised protrusions (HDMP) emerged from cracks in the calcified cartilage and subchondral bone. **c** Endochondral ossification of the high density mineralised protrusions resulting in subarticular osteophytes and superficial fibrillation of the articular cartilage. **d** The subchondral bone penetrates the articular cartilage. **e** Complete loss of the articular cartilage, and subchondral bone sclerosis

It is well known that marginal osteophytes grow by endochondral ossification with progressive changes of cellular proliferation, differentiation, and elaboration of intercellular matrix [21]. However, it is currently unknown how subarticular osteophytes form. A possible mechanism for the progression of subarticular osteophytes might be that they form as a consequence of microcracks in the calcified cartilage and subchondral bone [6], which might result in HDMP [11]. Supposing that HDMP is an early sign of the reactivation of the secondary ossification centre, the articular cartilage might be lost due to endochondral ossification as illustrated in Fig. 3, and not only as a consequence of fragmentation, wear and tear [7]. However, longitudinal studies are needed to investigate this hypothesis.

An important strength of the study is the use of systematic uniform random sampling. This sampling method ensures all positions in the complete human femoral heads are given equal probability of being sampled [18, 27]. Thereby, we did not selectively study the surface based on its specific appearance as normal or degrees of abnormalities but instead based on its random sampling. Another strength of the study is the separate quantitative estimates of osteophyte area. Because we used 2D histomorphometry, we were not able to measure the unbiased volume and surface area of individual discrete osteophytes in the femoral heads [28, 29]. However, the profile number together with the unbiased total osteophytic area and boundary length still indicate the degree of osteophytic formation for the individual femoral heads. A weakness of the current study is its cross-sectional design. Therefore, we can only speculate on how the subarticular osteophytes develop. Another weakness is that in areas devoid of articular cartilage, it was not possible to identify whether subarticular osteophytes were present or not. Thus, the estimates for the number of subarticular osteophytes in patients with OA were likely underestimated, while this was not the case for the control group. The control group was also slightly younger than the OA patients, although this difference was not statistically significant. Lastly, although the control group had a macroscopically normal femoral head, it cannot be excluded that the control group might have preclinical OA leading to the presence of histologically identifiable osteophytic changes. Still, the changes in the control group could be a degenerative phenomenon associated with age as we have also shown histomorphometrically for minor cartilage deterioration [20].

## Conclusions

In conclusion, the subarticular osteophytes were larger and more frequent in patients with osteoarthritis compared with the control group, suggesting that subarticular osteophytes are a common feature of OA pathology. Furthermore, in the control group, the frequency and size of subarticular osteophytes were more prevalent than expected, which may suggest that subarticular osteophytes might be an early degenerative phenomenon that might ultimately develop into clinical osteoarthritis. Consequently, subarticular osteophytes might possibly be used as an imaging marker for diagnosis and monitoring of early OA.

## Supplementary information

**Additional file 1.**

## Data Availability

The datasets generated during and/or analysed during the current study are available from the corresponding author on reasonable request.

## Abbreviations

HDMP: High Density mineralised protrusions
MRI: Magnetic resonance imaging
OA: Osteoarthritis
OARSI: Osteoarthritis Research Society International
WOMAC: Western Ontario, and McMaster Universities Arthritis Index
